# Boosted TCA cycle enhances survival of zebrafish to *Vibrio alginolyticus* infection

**DOI:** 10.1080/21505594.2017.1423188

**Published:** 2018-02-27

**Authors:** Man-Jun Yang, Zhi-Xue Cheng, Ming Jiang, Zao-Hai Zeng, Bo Peng, Xuan-Xian Peng, Hui Li

**Affiliations:** a Center for Proteomics and Metabolomics, State Key Laboratory of Biocontrol, Guangdong Province Key Laboratory for Pharmaceutical Functional Genes, School of Life Sciences, Sun Yat-sen University, University City, Guangzhou, People's Republic of China; b Tibet Vocational Technical College, Lhasha, People's Republic of China

**Keywords:** malate, metabolomics, reprogramming metabolomics, the TCA cycle, *Vibrio alginolyticus*, zebrafish

## Abstract

*Vibrio alginolyticus* is a waterborne pathogen that infects a wide variety of hosts including fish and human, and the outbreak of this pathogen can cause a huge economic loss in aquaculture. Thus, enhancing host's capability to survive from *V. alginolyticus* infection is key to fighting infection and this remains still unexplored. In the present study, we established a *V. alginolyticus*-zebrafish interaction model by which we explored how zebrafish survived from *V. alginolyticus* infection. We used GC-MS based metabolomic approaches to characterize differential metabolomes between survival and dying zebrafish upon infection. Pattern recognition analysis identified the TCA cycle as the most impacted pathway. The metabolites in the TCA cycle were decreased in the dying host, whereas the metabolites were increased in the survival host. Furthermore, the enzymatic activities of the TCA cycle including pyruvate dehydrogenase (PDH), α-ketoglutaric dehydrogenase (KGDH) and succinate dehydrogenase (SDH) also supported this conclusion. Among the increased metabolites in the TCA cycle, malic acid was the most crucial biomarker for fish survival. Indeed, exogenous malate promoted zebrafish survival in a dose-dependent manner. The corresponding activities of KGDH and SDH were also increased. These results indicate that the TCA cycle is a key pathway responsible for the survival or death in response to infection caused by *V. alginolyticus,* and highlight the way on development of metabolic modulation to control the infection.

## Introduction

Metabolomics provides a global view of endogenous metabolic patterns not only during cell growth, development and senescence, but also in response to genetic alterations and environmental factors [1,2]. Therefore, it has emerged as a powerful tool to explore metabolic processes, identify crucial metabolic biomarkers and reveal metabolic mechanisms [3]. Indeed, metabolomics is particularly useful in the study of environment-gene and -protein interactions [4–6], and the discovery of novel drugs [7,8]. More importantly, the unique features of metabolites, as compared to proteins and nucleic acids, lead to a novel approach to use metabolites to revert biological phenotypes like antibiotic resistance, which is termed as reprogramming metabolomics [3,9,10]. The reprogramming modulates the existing metabolome to an aimed metabolome [3], including that bacterial antibiotic-resistant metabolomes are reverted to the antibiotic-susceptible metabolomes [9,10], and host metabolomes sensitive to bacterial infection are restored to the metabolomes resistant to the infection [11–15]. Thus, metabolomics approach not only provides means for biomarker identification, but also helps in the screening of metabolic modulators to cope with adverse factors, including elevating bacterial sensitivity to antibiotics and host survival ability against bacterial infection.

*Vibrio alginolyticus* is a ubiquitous and halophilic Gram-negative bacterium found in temperate marine and estuarine environment. The pathogen has been associated with diseases not only in aquatic animals but also in humans, causing tissue damages in skin, ear and internal organs [16]. Vaccines and antibiotics are two ways to control infection caused by *V. alginolyticus*. Although vulnivaccine, a licensed vaccine against *V. vulnificus*, could protect eels from vibriosis for at least 6 months after vaccination by triple prolonged immersion at the glass eel stage [17], it is not convenient and economic to immunize small fishes in size, which are dominant fish species in aquaculture in China. The administration of antibiotics represents a simple and relative low-cost solution to bacterial infections. However, abuse and misuse of antibiotics lead to the emergence of multidrug-resistant bacteria, microorganism substitution, ecological and public health impacts [18–20]. Therefore, it is required to develop low-cost and “green” approaches for controlling the infection caused by *V. alginolyticus.*

Here GC-MS based metabolomics has been used to analyze metabolic profile in humoral fluid of zebrafish exposed to *V. alginolyticus* V12G01, leading to survival- and dying-associated metabolomes. Zebrafish is an animal model for studies of microbial infection [21–23]. Instead of using kidney leukocytes, we used humoral fluid because it represents a global metabolic status to the infection and provides enough samples for analysis. We found that it is required to enhance the TCA cycle for the survival of these animals. Further results showed that exogenous malate increases zebrafish survival by 30–40%. These findings are very important in understanding the anti-infective mechanisms in metabolic regulation.

## Results

### Metabolomic profiling of humoral fluid of zebrafish to V. alginolyticus infection

**Figure 1.**
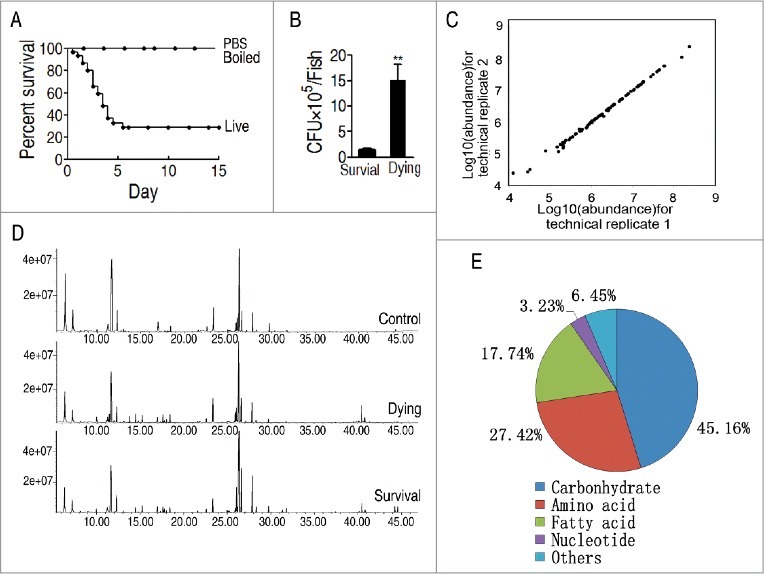
Metabolomic profiling of *D.* *rerio* humoral fluid. A, Percent survival of *D. rerio* infected with PBS (control 1, PBS), boiled *V. alginolyticus* (8 × 10^5^ CFU/fish) (control 2, boiled), and sublethal dose of *V. alginolyticus* (8 × 10^5^ CFU/fish) (live). B, Bacterial number of survival and dying fish after bacterial challenge. C, Reproducibility of metabolomic profiling platform. Metabolite abundances quantified in cell samples over two technical replicates are shown. Correlation coefficient between technical replicates varies between 0.990 and 0.999. This plot shows the two replicates with the weakest correlation of 0.990. D, Representative total ion current chromatogram from control (saline), dying and survival samples. E, Category of the identified metabolites.

To investigate the metabolic features of zebrafish, *Danio rerio*, to *V. alginolyticus* infection, these fish were infected with sub-lethal dose of *V. alginolyticus* V12G01 (8 × 10^5^ CFU/fish), which caused approximately 70% death, as experimental group, and injected with either the same volume of phosphate buffer saline (PBS) or the same amount of boiled bacterial cells dissolved in the same volume, which led to zero death, as control groups (Fig. 1A). Humoral fluid was immediately collected as dying group when *D. rerio* showed the symptoms of death, whereas the humoral fluid of *D. rerio* survived from the infection was collected as survival group. Meanwhile, the same approach was used for collection of the humoral fluid from the group with the injected saline solution as control. In addition, bacterial counting was carried out in mixture of fish organs, liver, spleen and pancreas. Bacterial amounts were tenfold higher in the dying fish than the survival fish (Fig. 1B), no bacteria grew in the control. Ten individuals with two technical repeats were carried out in each group, yielding a total of 60 data sets. The correlation coefficient between technical replicates varied between 0.990 and 0.999, demonstrating the reproducibility of the data (Fig. 1C). A total of 240 aligned individual peaks were obtained from each sample (Fig. 1D). After removal of internal standard ribitol and any known artificial peaks, 64 metabolites were identified. Among them, 45.16%, 27.42%, 17.74%, 3.23%, 6.45% were categorized to carbohydrate, amino acid, fatty acid, nucleotide and others, respectively (Fig. 1E).

### Differential metabolomics responsible for the survival and the dying

**Figure 2.**
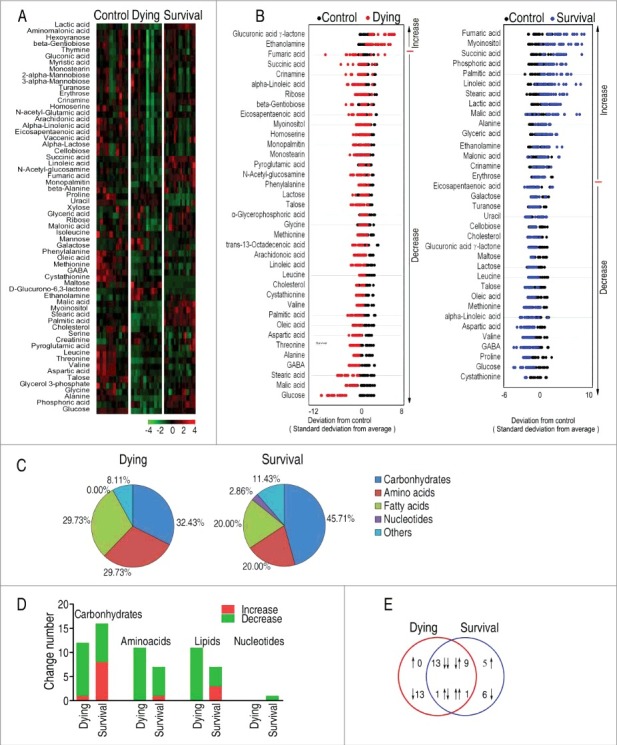
Differential metabolomic profiling between dying and survival groups in response to *V.* *alginolyticus* infection compared with control. A, Heat map showing differential metabolites. Green color and red color indicate increase and decrease of metabolites relative to the median metabolite level, respectively (see color scale). B, Z-score plot of differential metabolites based on control. The data of dying (left) and survival (right) groups were separately scaled to the mean and standard deviation of control. Each point represents one metabolite in one technical repeat and colored by sample types. C, Category of identified metabolites of differential abundance. D, Number of differential abundance of metabolites. E, Venn diagram for comparison of differential metabolites between the dying and survival groups.

To explore the metabolic biomarkers that distinguish the survival from the dying *D. rerio*, a two-sided Wilcoxon rank-sum test coupled with permutation test was used to identify the differential abundance of metabolites of survival group to control group, and dying group to control group. Thirty-six and thirty-seven metabolites were identified (p < 0.05) in the survival group and the dying group, respectively, which corresponded to false discovery rate (FDR) of 0.0742% and 0.5956%. The identified metabolites are shown in Fig. 2A as heatmap, where the three groups, dying, survival and control groups, are clearly separated. In addition, Z-score plot spans from −4.24 to 8.30 in the survival group and from −10.95 to 6.22 in the dying group. In comparison to the control group, two metabolites were increased and thirty-five metabolites were decreased in the dying group, and sixteen metabolites were increased and twenty metabolites were decreased in the survival group (Fig. 2B). Among the thirty-six metabolites and thirty-seven metabolites, 45.71% and 32.43% were carbohydrates; 20.00% and 29.73% were amino acids; 20.00% and 29.73% were fatty acids; 2.86% and 0% were nucleotides; and 11.43%, and 8.11% were other metabolites, respectively (Fig. 2C). Among the differential abundance of metabolites in number, carbohydrates and lipids became significantly lower in the dying group than the survival group (Fig. 2D). There were twenty-four metabolites shared between the two groups, where thirteen metabolites (eicosapentaenoic acid, cholesterol, lactose, leucine, talose, oleic acid, methionine, linoleic acid, aspartic acid, valine, GABA, glucose and cystathionine) were decreased, and one metabolite (ethanolamine) was increased in both groups. Of notice, nine metabolites (fumaric acid, myoinositol, succinic acid, palmitic acid, linoleic acid, stearic acid, malic acid, alanine and crinamine) were decreased in the dying group but increased in the survival group, while one metabolite (glucuronic acid γ-lactone) was increased in the dying group but decreased in the survival group. There were thirteen decreased metabolites (glycerophosphoric acid, glycine, pyroglutamic acid, monostearin, phenylalanine, homoserine, ribose, gentiobiose, 13-Octadecenoic acid, arachidonoic acid, N-Acetyl-D-glucosamine, monopalmitin and threonine) only present in the dying group, and eleven metabolites, including five (malonic acid, erythrose, phosphoric acid, glyceric acid and lactic acid) increased metabolites and six (proline, maltose, galactose, turanose, uracil and cellobiose) decreased metabolites, only present in the survival group (Fig. 2E). Thus, these results indicate that the surviving and dying *D. rerio* have distinct metabolic profiles.

### Identification of key metabolites associated with the survival

**Figure 3.**
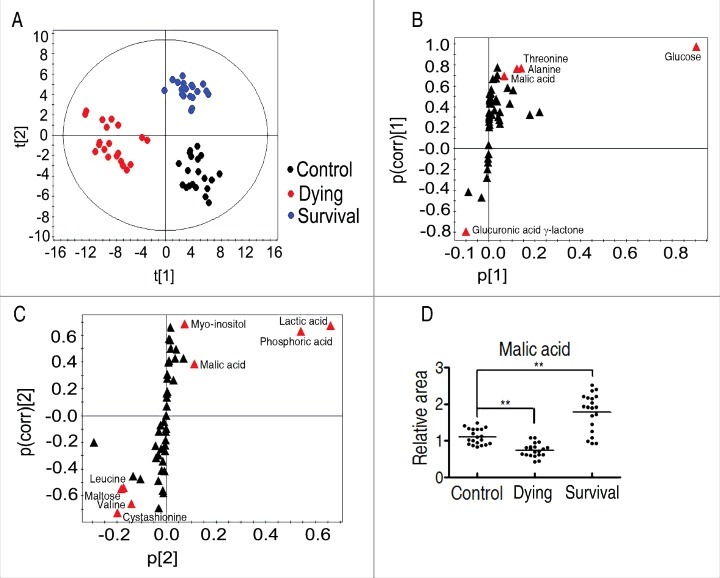
Identification of crucial metabolites. A, The PCA analysis of the control group, the dying group and the survival group. Each dot represents the technique replicates in the plot. t[1] and t[2] explain 98.7% of the total variance which allows confident interpretation of the variation. B and C, S-plot generated from OPLS-DA. Predictive component p[1] and correlation p(corr)[1] differentiate the survival from the control and the dying. Predictive component p[2] and correlation p(corr)[2] separate the control group from the dying group. Triangle represents individual metabolite, where potential biomarkers are highlighted with red, which is greater or equal to 0.05 and 0.5 for absolute value of covariance p and correlation p(corr), respectively. D, Comparison of the crucial biomarker malic acid among the control, the survival and the dying groups.

To explore the most crucial metabolites related to *D. rerio* survival from infection, orthogonal partial least square discriminant analysis (OPLS-DA) was conducted to recognize the sample pattern, and the three groups were seperated. Component t[1] separated the survival group and the control group from the dying group, while component t[2] differentiated survival from control as well as variables within the dying group (Fig. 3A). Discriminating variables are shown by S-plot (Fig. 3B and C) where cut-off values are set as greater or equal to 0.05 and 0.5 for absolute value of covariance p and correlation p(corr), respectively. Crucial biomarkers were selected from component p[1] and p[2], and the biomarker should be present in both of the components based on the two S-plots. Malic acid is thus identified, whose abundance is significantly increased in the survival group than the dying and control groups (Fig. 3D).

### The change of the TCA cycle is associated with survival and dying fish

**Figure 4.**
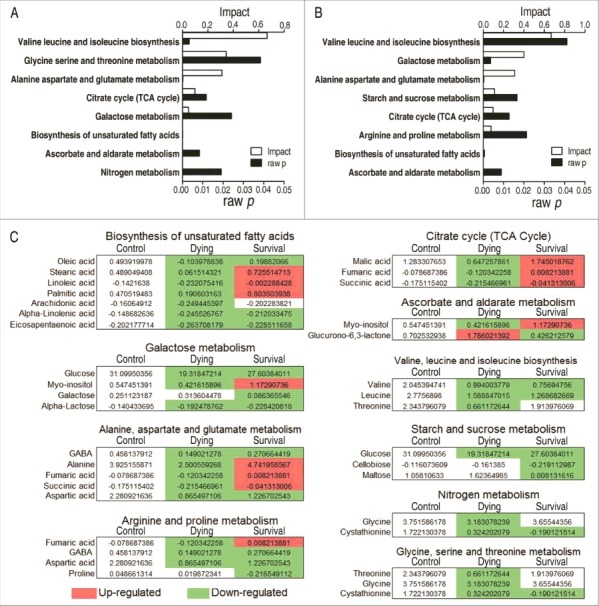
Pathway enrichment. A and B, Pathway enrichment of varied metabolites in the dying group (A) and in the survival group (B). Significant enriched pathways are selected to plot. C, Integrative analysis of metabolites in significantly enriched pathways. Red color and green color indicate increased and decreased metabolites, respectively.

Enriched metabolic pathways are especially important for understanding the metabolome alterations during *V. alginolyticus* infection. The differential metabolites identified in the survival group or the dying group were separately analyzed. Eight metabolic pathways were separately enriched in the survival and dying groups. Among them, six metabolic pathways (valine, leucine and isoleucine biosynthesis, alanine, aspartate and glutamine metabolism, the TCA cycle, galactose metabolism, biosynthesis of unsaturated fatty acids, and ascorbate and aldarate metabolism) were shared in both groups, while glycine, serine and threonine metabolism, and nitrogen metabolism were only present in the dying group, and starch and sucrose metabolism, arginine and proline metabolism were determined only in the survival group (Fig. 4A and B). Besides the metabolite abundance of valine, leucine and isoleucine biosynthesis, and galactose metabolism, which was decreased in both of the survival and dying groups, the metabolite abundance of alanine, aspartate and glutamine metabolism, biosynthesis of unsaturated fatty acids, and ascorbate and aldarate metabolism was reduced in all metabolites of the dying group but in half metabolites of the survival group. Of particular interest is the metabolites of the TCA cycle that were decreased in the dying group but increased in the survival group (Fig. 4C).

**Figure 5.**
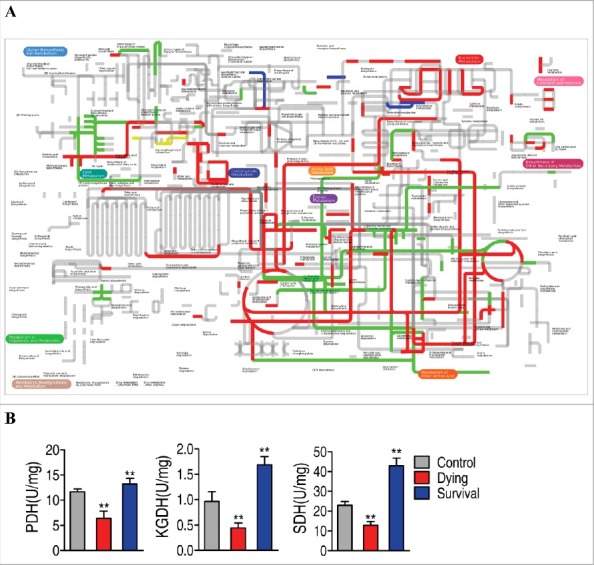
Comparative metabolic pathway analysis and enzyme activity measurement between the dying group and survival group. A, Analysis of the metabolic profiles resulting from *D. rerio* challenged with *V. alginolyticus* provides a better insight into the effects of 46 significant metabolites (*p*<0.05). Based on the KEGG compound (http://www.kegg.jp/kegg/compound/), metabolic network pathways in *D. rerio* are further analyzed with iPath2.0 (http://pathways.embl.de/iPath2.cgi). Red line represents increase in the survival group but decrease in the dying group; blue line represents increase in the dying group but decrease in the survival group; yellow line represents increase in both of the dying group and survival group; green line represents decrease in both dying group and survival group. B, Activity of PDH, KGDH and SDH in the control group, the dying group and the survival group.

Moreover, comparative metabolic pathway analysis between the survival group and the dying group was carried out in iPath [24]. The resulting global overview map provides a better insight into the effects of the bacterial pathogen on metabolism of the fish, where red line represents increased pathways in the survival group but decreased in the dying group; blue line represents increased pathways in the dying group but decreased in the survival group; yellow and green lines represent increased or decreased pathways in both groups, respectively. We identified elevated carbohydrate metabolism as well as elevated production of key biomass components, and decreased alanine, aspartae and glutamate meatbolism as the associated metabolic pathways (Fig. 5A), implying the importance of the activation of the TCA cycle. To validate the contributing role of the TCA cycle in the fish survival upon infection, the activity of three key enzymes in the central carbon metabolism of zebrafish was measured, including PDH that transforms pyruvate into acetyl-CoA, KGDH that converts α-ketoglutarate to succinyl-CoA, and SDH that catalyzes the oxidation of succinate to fumarate. Activity of the three enzymes was decreased and elevated in the dying group and the survival group, respectively (Fig. 5B). These results are consistent with the metabolic analysis that the TCA cycle response to the bacterial infection contributes to the outputs, dying or survival. Thus, these results suggested that TCA cycle could be a key component in defending *V. alginolyticus* infection.

### Exogenous malate potentiates zebrafish against V. alginolyticus infection

**Figure 6.**
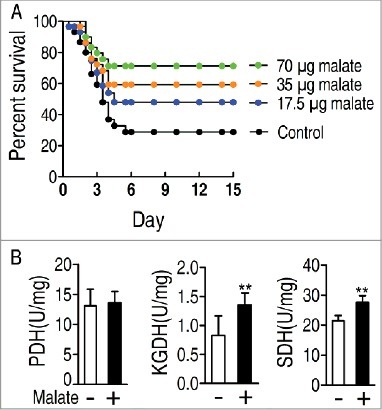
Percent survival of *D. rerio* challenged with *V. alginolyticus* in the presence or absence of the indicated dose of malate (A). Activity of PDH, KGDH and SDH in the presence or absence of exogenous malate (B).

Crucial biomarkers can reprogram the existed metabolome to an aimed metabolome to defense bacterial infection through exogenous administration [3]. Malic acid, a key metabolite in TCA cycle, is identified as the crucial biomarker (Fig. 3D). We speculated that elevated abundance of malic acid may be required for zebrafish survival from *V. alginolyticus* infection. To test this idea, *D. rerio* were injected with exogenous malate followed by challenge with *V. alginolyticus.* Exogenous malate elevated the survival of *D. rerio* in a dose-dependent manner, where the survival rate was increased from 25.0% to 72.1% when 70 μg malate per zebrafish was used (Fig. 6A). These results indicate that exogenous malate is an effective modulator in *D. rerio* against *V. alginolyticus* infection. For further demonstrating that the action of exogenous malate was attributed to promote the TCA cycle, the activity of PDH, KGDH and SDH was detected in the presence of malate. Exogenous malate promoted activity of KGDH and SDH, but not that of PDH (Fig. 6B).

### Discussion

Metabolites are recognized as important modulators in biological processes in recent years such as immunoregulators that modulate hosts' ability against bacterial infections [25–29]. *V. alginolyticus* is as an important pathogen that infects both humans and different fish species. Besides the unavailability of effective vaccines against *V. alginolyticus*, the overuse of antibiotics to treat bacterial infections nevertheless leads to the global spread of antibiotic-resistant bacteria [18,19]. Thus, there is an urgent need to develop novel strategies to treat bacterial infections. To take the advantage of our recently developed reprogramming metabolomics [3], we adopted GC-MS based metabolomics to characterize the differential metabolome responsible for host survival, and identify key metabolic pathways and crucial biomarkers. By this strategy, we came to the findings that TCA cycle is the most important pathway for host survival, and malic acid is the most crucial biomarker. Exogenous malate greatly elevates the survival of zebrafish infected by *V. alginolyticus*, indicating that this metabolite reprograms the dying-associated metabolome to the survival-associated metabolome and thereby reverts the consequence of infection. A possible mechanism may be attributed to the event that elevated crucial metabolites such as succinate promote innate immune response, which is supported by induction of succinate to IL-1β through hypoxia inducible factor-1α [26]. Logically, elevated malate in downstream reduces the use of succinate in upstream. Recently, we showed that glucose reprograms anti-infective metabolome with promoting unsaturated fatty acid biosynthesis as a characteristic feature to cope with infection in tilapia [30]. L-Leucine-induced anti-infective metabolome to eliminate *Streptococcus iniae* in tilapia [31]. Elevated myo-inositol or L-Valine promotes phagocytosis to eliminate antibiotic-resistant bacterial pathogens in mice [12,27]. Taken together, metabolome reprogramming is a useful low-cost and “green” approach to combat bacterial pathogens.

Bacterial infection induces host metabolic changes like central carbon metabolism, amino acid metabolism and fatty acid metabolism [32,33]. In the present study, a total of eight metabolic pathways were separately enriched in both of the survival group and the dying group. The abundance of the metabolites of the eight metabolic pathways was different in the three groups. Of particular interest is the TCA cycle that forms a characteristic feature between the dying-associated metabolome and the survival-associated metabolome, being decreased and elevated in the dying-associated metabolome and the survival-associated metabolome, respectively, which was further highlighted by iPath and validated by detection of enzyme activity. These results indicate that the status of the TCA cycle is a biomarker for the consequences of fish infected with *V. alginolyticus*, survival or death. Thus, the TCA cycle is a key pathway in zebrafish to fight against *V. alginolyticus*. Additionally, we have shown that elevated glucose is a characteristic feature in the survival tilapias infected with *Edwardsiella tarda.* Exogenous glucose reprograms tilapia metabolome that increases their survival to *E. tarda* infection, characterizing with elevated stearic acid and palmitic acid and attenuated TCA cycle [11,30]. These data indicate that crucial metabolites that revert the metabolome are related to host and bacterial species.

Accordantly, malic acid in the TCA cycle plays a main role not only in distinguishing the dying group from the survival and the control groups, but also in separating the control from the survival group. Therefore, it is identified as a crucial metabolite. In the TCA cycle, malic acid is an intermediate formed by the addition of an -OH group on the *si* face of fumarate, and is transformed to oxaloacetate by malate dehydrogenase. In addition, it can also be formed from pyruvate via anaplerotic reactions [34]. Lower abundance and higher abundance of malic acid is detected in the dying and survival groups than the control, respectively, further supporting the above conclusion that the TCA cycle is a key pathway deciding survival or death in response to infection caused by *V. alginolyticus.*

These results on the TCA cycle as the key pathway and the malic acid as the crucial metabolite motivated us to reprogram the TCA cycle in the dying-related metabolome to that in the survival-related metabolome through exogenous administration of malate. Elevated survival is detected in the malate-induced zebrafish in a dose-dependent manner, which is supported by elevated KGDH and SDH in the presence of exogenous malate. These results further demonstrate the importance of enhancing the TCA cycle in resistance to *V. alginolyticus* infection.

As described earlier, we selected zebrafish as an animal model because it has been used in exploring anti-infective immunity against *Vibrio* species as the fish model [21–23]. *Vibrio* species including *V. alginolyticus* are marine bacteria. Therefore, it would be interesting to use marine Medaka as a fish model to investigate the issue. Meanwhile, the zebrafish used were not genetically inbred, and thereby there are numerous genetic factors that could underlie the difference in resistance to *V. alginolyticus* infection. Although the subsequent data on the protective effect of malate can effectively validate our findings, genetically inbred zebrafish are recommended. In addition, the humoral fluid was squeezed out of the fish by high centrifugation force and should contain a mixture of blood plasma and cell lysate. The ratio of these components was in turn dependent on the degree of necrosis in the fish at the time of collection, which may have contributed to the data. Because the degree of necrosis was associated with disease severity, it is suggested that the metabolic difference between the survival and dying fish may be related to the use of the humoral fluid as a sample.

To conclude, our results indicate that boosted TCA cycle enhances survival of zebrafish to *V. alginolyticus* infection, which may be attributed to providing increased energy against the infection. Further investigations will focus on the following. 1) To reprogram the current metabolome to enhance host's immunity, which requires further functional metabolome for analysis of metabolic modulation and exploration of the modulation with the cytokine expression; 2) To investigate whether the elevated malate itself has antibacterial effects; 3) To explore interaction between metabolite and proteins. The molecular mechanisms involved in pathogen interference in the TCA cycle wait investigation. These metabolites and their reprogramming mechanisms are dependent on bacteria species and hosts. Thus, elucidation of the interaction between the two would benefit our idea for development of novel method to boost host's immunity to cope with bacterial invasions in a vaccine- and antibiotic-independent manner.

## Materials and methods

### Bacterial strain, zebrafish and culture conditions

*V. alginolyticus* V12G01 (GenBank accession Nos. AAPS01000007), isolated from Plum Island Ecosystem-LTER, USA, from surface waters by plating, was used in this study. A single colony was cultured in Luria-Bertani (LB) medium (1% w/v peptone, 0.5% w/v yeast extracts, 1% w/v NaCl, pH7.4) at 30°C overnight in a shaker bath as seed. Fresh overnight cultures were inoculated into LB medium. The bacteria were cultured at 30 ^0^C and grown to an OD600 of 1.0. *D. rerio* about 3 months (body length: 2.8 to 3.2 cm, body weight: 0.28 to 0.31 g) were purchased from a local commercial market in Guangzhou. These animals were acclimated in laboratory environment for two weeks and were demonstrated to be free from *Vibrio* species by bacteriology before using in subsequent experiment. The rearing and treatment of the experimental fish were approved by Sun Yat-sen University.

### Bacterial infection and fish symptoms

*V. alginolyticus* was cultured in LB medium at 30 °C and grown to an OD600 of 1.0. The cultures were centrifuged and diluted using sterile saline until 1.0 of OD600. LD_50_ dose was determined by intraperitoneal injection into zebrafish. In brief, 10 fish for each dose were challenged by 5 μL PBS with series of doses ranging from 1 × 10^5^ to 1 × 10^6^ CFU/fish. The LD_50_ was determined as 6 × 10^5^ CFU/fish. Then, 8 × 10^5^ CFU of live and boiled *V. alginolyticus* were dissolved in 5 μL PBS as experimental and control groups, respectively, to challenge every fish of the two groups. Some of the fish started to show infectious symptoms after 40 h and died within 60 h. *V. alginolyticus* was isolated from the diseased fish for validation of the infection through bacterial counting in *Vibrio* selective agar (TCBS agar) using mixture of fish liver, spleen and pancreas homogenate. The remaining infected fish survived without visible symptoms.

### Metabolomics analysis

#### Sample preparation

Zebrafish body fluid was collected as previously described with a few modifications [14]. In brief, zebrafish were rinsed with distilled water and then wiped thoroughly with sterilized gauze. These animals were cut into five pieces on ice and then weighted. The appropriate volume of saline (100 μL/100 mg) was added according to the weight. After centrifugation at 3000 × g, 4 ^0^C, 100 μL fluid was isolated for the further study of metabolites. Metabolites were extracted with 0.2 mL cold methanol (Sigma) containing 10 μL 0.1 mg/mL ribitol (Sigma) as an analytical internal standard. After centrifugation at 12,000 × g for 10 min, the supernatant was concentrated in a rotary vacuum centrifuge device (LABCONCO). The dried polar extracts were used for GC-MS analysis.

#### GC-MS analysis

GC-MS analysis was carried out with a variation on the two-stage technique [14]. In brief, samples were derivatized and then used to firstly protect carbonyl moieties through methoximation, through a 90 min, 37⁰C reaction with 40 μL of 20 mg/mL methoxyamine hydrochloride (Sigma-Aldrich) in pyridine, followed by derivatization of acidic protons through a 30 min, 37⁰C reaction with the addition of 80 μL N-methyl-N-trimethylsilyltrifluoroacetamide (MSTFA, Sigma-Aldrich). The derivatized sample of 1 μL was injected into a 30 m × 250 μm i.d. × 0.25 μm DBS-MS column using splitless injection and analysis was carried out by Agilent 7890A GC equipped with an Agilent 5975C VL MSD detector (Agilent Technologies). The initial temperature of the GC oven was held at 85⁰C for 5 min followed by an increase to 270⁰C at a rate of 15⁰C min^−1^ and then held for 5 min. Helium was used as carrier gas and flow was kept constant at 1 mL min^−1^. The MS was operated in a range of 50–600 m/z.

#### Data Processing

Spectral deconvolution and calibration were performed using AMDIS and internal standards. A retention time (RT) correction was performed for all the samples, and then the RT was used as reference against which the remaining spectra were queried and a file containing the abundance information for each metabolite in all the samples was assembled. Metabolites from the GC-MS spectra were identified by searching in National Institute of Standards and Technology (NIST 08) Mass Spectral Library. Among the detected peaks of all the chromatograms, 265 peaks were considered as endogenous metabolites excluded internal standard ribitol. The resulting data matrix was normalized by the concentrations of added internal standards and the total intensity. The resulting normalized peak intensities formed a single matrix with Rt-m/z pairs for each file in the dataset. This file was then used for subsequent bioinformatics analyses.

#### Bioinformatics analyses

Data transformations and manipulations were done using Excel. Multivariate statistical analysis was performed with SIMCA-P (Umetrics). Hierarchical clustering was performed on the log transformed normalize date, and completed in the R platform with the package gplots (http://cran.r-project.org/src/contrib/Descriptions/gplots.html) using the distance matrix. Prior to analysis, sets of metabolites data subtracted the median metabolites and were scaled by the quartile range in the sample. Z-score analysis scaled each metabolite according to a reference distribution, and calculated based on the mean and standard deviation of reference sets a control. Simca-p was used to perform sample pattern recognition. OPLS-DA was performed using centred scaling. Detailed accounts of pattern recognition methods were previously described [31]. SPSS 13.0 and Prism v5.01 (GraphPad, La Jolla, CA, USA) were used to draw the histogram the scatter plot. Comparative metabolic pathway analysis between the death group and the survival group was performed using iPath2.0 [24].

### Exogenous addition of malate and bacterial challenge

Administration of exogenous malate was performed as previously described [29,35]. In brief, acclimatized zebrafish were randomly divided into four groups, twenty each. Out of the four groups, three were used for test groups and one was for control group. Fish in the three test subgroups were administrated by intraperitoneal injection with 17.5, 35 or 70 μg malate, which was designed by preliminary examination, dissolved in 5 μL PBS, once daily for 3 days. These fish showed normal growth and no any abnormal syndrome was found. The control was injected with same volume of PBS only. On the third day, these zebrafish were challenged by intraperitoneal inoculation of 8 × 10^5^ CFU/fish *V. alginolyticus*. The zebrafish were observed twice daily for 15 days.

### Measurement of activity of pyruvate dehydrogenase (PDH), α-ketoglutaric dehydrogenase (KGDH) and succinate dehydrogenase (SDH)

Measurement of activity of PDF, KGDH and SDH was carried out as previously described [29]. Zebrafish were acclimatized for two weeks to laboratory conditions before subsequent experiment. One hundred and twenty zebrafish were randomly divided into 4 groups, thirty each. Group 1 was intraperitoneally injected with sterile water as control; Group 2 was intraperitoneally injected with 8 × 10^5^ CFU *V. alginolyticus* /fish, and then was divided to dying and survival groups. These fish were collected at 48 h. Groups 3 and 4 were intraperitoneally injected with 70 μg malate and the same volume of sterile water daily, respectively, for 3 days and then collected. All fish, trimmed of head and visible fat surrounding around abdomen, were broken by sonication for 5 min at a 200 w power in cold PBS, following by centrifugation at 12,000 rpm for 10 min to remove insoluble material. Supernatant containing 400 μg or 200 μg total proteins of the whole fish tissues was transferred to the PDH and KGDH reaction mix (0.5 mM MTT, 1 mM MgCl_2,_ 6.5 mM PMS, 0.2 mM TPP, 50 mM PBS and 2 mM sodium pyruvate for PDH or 2 mM sodium α-ketoglutaric for KGDH) or SDH reaction mix (0.5 mM MTT, 13 mM PMS, 5 mM succinate, 50 mM PBS) to a final volume of 200 μL in 96-well plate. Subsequently, the plate was incubated at 37^0^C for 30 min for PDH and KGDH, while 10 min for SDH, and then each plate was measured at 566 nm for colorimetric reading.
